# Novel bioactive compounds in the treatment of osteoporosis: chemistry-driven discovery from preclinical validation to clinical translation

**DOI:** 10.3389/fchem.2026.1917991

**Published:** 2026-09-18

**Authors:** Honghui Fan, Songtao Jin

**Affiliations:** 1 Department of Orthopedics, Shaoxing People’s Hospital, Shaoxing, Zhejiang, China; 2 School of Medicine, Shaoxing University, Shaoxing, Zhejiang, China

**Keywords:** bone remodeling, clinical translation, drug delivery, medicinal chemistry, natural products, osteoporosis

## Abstract

Osteoporosis affects an estimated 200 million people worldwide. Fragility fractures of the hip, spine, and wrist carry high mortality and impose a heavy socioeconomic burden. Bisphosphonates, denosumab, selective estrogen receptor modulators (SERMs), teriparatide, abaloparatide, and the recent sclerostin antibody romosozumab have improved clinical outcomes, but unmet needs remain. Long-term antiresorptive use is complicated by atypical femoral fractures and osteonecrosis of the jaw; denosumab discontinuation causes rebound bone loss; romosozumab carries a cardiovascular caution. Persistence on therapy beyond 1 year is low, and no oral osteoanabolic agent has reached the clinic. Against this background, medicinal chemistry is producing a wave of next-line compounds. These include rationally designed small molecules and peptidomimetics targeting the Wnt pathway, calcium-sensing receptor antagonists (calcilytics) intended to mimic pulsatile parathyroid hormone, refined cathepsin K inhibitors, modulators of activin and myostatin signaling, and bone-targeted nanomedicines. Plant-derived scaffolds with anti-osteoporotic activity, particularly icariin, icaritin, genistein, naringin, resveratrol, curcumin, oleanolic acid, psoralen, and berberine, are being optimized chemically to address poor bioavailability. Senolytic combinations and PROTAC-based degraders represent newer modes of action. This review examines the chemistry behind these compounds, their structure–activity relationships, their preclinical pharmacology, and their progress through clinical evaluation. We pay particular attention to the failures (odanacatib, calcilytics, integrin antagonists, Src inhibitors) as well as the successes, because the lessons from attrition shape the chemistry choices that follow. We attempt to identify those chemistry-led strategies that have most reliably accelerated bench-to-bedside translation in bone disease, and those that have not.

## 1 Introduction

Osteoporosis is the most prevalent metabolic bone disease worldwide. The operational definition is a bone mineral density (BMD) more than 2.5 standard deviations below the young adult mean (T-score ≤ −2.5), or the occurrence of a low-trauma fragility fracture (Kanis et al., 1994). One in three women and one in five men older than 50 will sustain an osteoporotic fracture in their remaining lifetime (Johnell and Kanis, 2006). Hip fractures alone carry a 1-year mortality of 20%–30% and are a leading cause of disability in older adults (Cooper et al., 1993). The annual cost of osteoporosis in the United States is projected to exceed USD 25 billion by 2025, with comparable burdens in Europe and Asia (Burge et al., 2007). The disease reflects an imbalance between bone resorption and bone formation, together with changes in bone properties, microarchitecture, remodeling dynamics, endocrine regulation, and aging-related cellular dysfunction. Current drug therapy has certain degree of clinical value, but it does not fully solve the long-term need for safe, effective, accessible, and mechanism-based skeletal protection.

Pharmacological options divide broadly into antiresorptive and osteoanabolic agents (Compston et al., 2019). The nitrogen-containing bisphosphonates (alendronate, risedronate, ibandronate, zoledronic acid) and the anti-RANKL antibody denosumab dominate the antiresorptive class (Lacey et al., 2012; Russell et al., 2008). Raloxifene and bazedoxifene retain a role in postmenopausal management (Ettinger et al., 1999). Bone formation can be stimulated by the parathyroid hormone (PTH) analogues teriparatide and abaloparatide, while romosozumab, a humanized anti-sclerostin monoclonal antibody, combines anabolic and antiresorptive activities by neutralizing sclerostin and uncoupling formation from resorption (McClung et al., 2014; Neer et al., 2001). Adverse events continue to constrain clinical use. Long-term bisphosphonate or denosumab exposure can produce atypical femoral fractures and osteonecrosis of the jaw (Khan et al., 2015; Shane et al., 2014). Denosumab discontinuation is followed by a rapid rebound in bone turnover with documented multiple vertebral fractures within 12 months. Romosozumab carries a cardiovascular signal that led to regulatory cautions. Adherence rates beyond 12 months remain disappointing (Hiligsmann et al., 2019).

These shortcomings have driven a sustained chemistry-led search for compounds with better efficacy, safety, and patient acceptability. Several categories are now in active development. Orally bioavailable small molecules are being designed against osteocyte signaling, including sclerostin loop2 mimetics and Wnt agonists. Cathepsin K inhibitors have been re-engineered for improved selectivity, exemplified by MIV-711. Calcium-sensing receptor antagonists are being pursued for their pulsatile PTH-like effect. Structurally diverse natural products are being optimized for bioavailability (Khosla and Hofbauer, 2017; Rachner et al., 2011). In parallel, bone-targeted delivery systems based on bisphosphonate conjugation, tetracycline tethering, and oligopeptide-functionalized nanoparticles aim to direct compounds preferentially to mineralized surfaces (Carbone et al., 2017). Newer modes of action including targeted protein degradation (PROTACs), molecular glues, and senolytic combinations open therapeutic options that did not exist a decade ago (Burslem and Crews, 2020).

The translational gap between preclinical promise and clinical performance is wide. Several candidates with compelling rodent pharmacology have failed in late-phase development. Odanacatib, a cathepsin K inhibitor, was withdrawn because of a numerically increased stroke rate despite efficacy. Calcilytics did not match alendronate in head-to-head studies. Saracatinib and the integrin αvβ3 antagonists did not progress beyond early bone trials (Murphy et al., 2005; Stoch and Wagner, 2008). Bridging the gap requires an integrated pipeline in which medicinal chemistry, structural biology, pharmacology, and clinical-trial design feed back into one another.

This review surveys the contemporary pipeline of bioactive compounds for osteoporosis with an emphasis on chemistry. We outline the molecular targets that underpin rational design. This article reviews the established therapies and their limitations. Following an overview of foundational concepts, the discussion progresses to synthetic osteoanabolics, antiresorptive small molecules, and natural-product-derived compounds, with detailed analyses of their structure–activity relationships and clinical status. Attention then shifts to senolytic strategies aimed at combating bone aging. Subsequent sections explore cutting-edge bone-targeted delivery technologies and delineate the critical translational hurdles currently facing the field. The review culminates in an exploration of future paradigms, including AI-guided discovery, PROTAC degraders, and personalized regimens. Throughout, we underscore how pivotal chemistry choices have dictated both the successes and failures of these therapeutic ventures (Figure 1).

**FIGURE 1:**
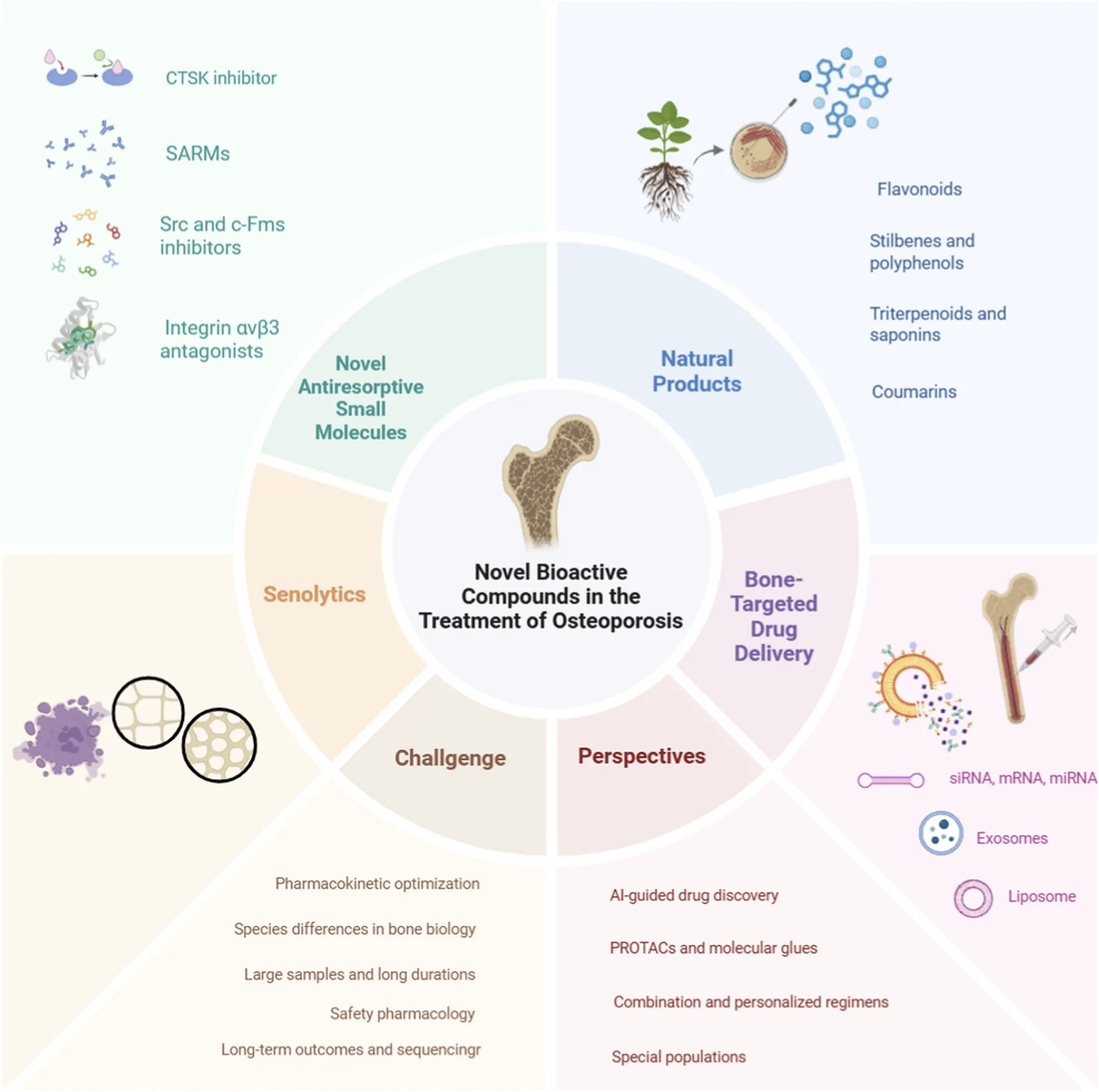
Novel bioactive compounds in the treatment of osteoporosis. Types of drugs developed for osteoclasts and osteoblasts during bone remodeling, along with their translational challenges and future directions.

Unlike many osteoporosis reviews that focus primarily on clinical management, bone biology, epidemiology, or single therapeutic classes, this review adopts a chemistry-centered perspective on bioactive compounds for osteoporosis. We integrate molecular targets, scaffold design, structure-activity relationships, pharmacokinetic constraints, bone-targeted delivery, translational barriers, and emerging discovery strategies into a single therapeutic roadmap. This organization is intended to help readers understand not only which compounds have been investigated, but also why specific chemical features have contributed to therapeutic success, limited efficacy, or development failure. While the discussion is relevant to osteoporosis in general, particular attention is given to postmenopausal and age-related osteoporosis, because these settings account for a major proportion of clinical disease burden and are closely linked to changes in bone remodeling, cellular senescence, and long-term treatment needs. Where evidence relates primarily to glucocorticoid-induced osteoporosis or other secondary forms, this should be specified in the relevant section.

**Table:** Key Terms.

Term	Definition
Osteoanabolic therapy	A therapeutic strategy that increases bone formation, usually by stimulating osteoblast lineage activity or bone-forming signaling pathways
Antiresorptive therapy	A therapeutic strategy that reduces bone loss by inhibiting osteoclast formation, survival, or resorptive function
Senolytic	A compound or compound combination designed to eliminate senescent cells or reduce senescence-associated tissue dysfunction
Bone-targeted delivery	A delivery strategy that uses bone-seeking ligands or carriers to increase drug accumulation at bone surfaces or in the bone microenvironment
PROTAC	A proteolysis-targeting chimera that induces degradation of a selected protein, typically through proximity-based recruitment of an E3 ligase
Network pharmacology	A systems-level approach that analyzes compounds, targets, and pathways as interconnected networks rather than isolated one-drug-one-target relationships

**Table:** Comparative overview of major bioactive compound classes for osteoporosis.

Compound	Target	Mechanism	Chemistry feature	Evidence stage	Advantages	Limitations	Clinical relevance
Bisphosphonates	Hydroxyapatite and osteoclasts	Suppress osteoclast-mediated bone resorption	P-C-P scaffold with high mineral affinity	Approved	Durable anti-fracture evidence and strong skeletal retention	Long skeletal residence, adherence issues, rare atypical fracture or osteonecrosis concerns	First-line antiresorptive option for many patients
RANKL inhibition	RANKL-RANK signaling	Reduces osteoclast differentiation and activity	Antibody-based biologic rather than small molecule	Approved	Potent reversible antiresorptive effect	Requires continuous dosing strategy and transition planning	Useful in high-risk patients requiring strong antiresorptive therapy
PTH/PTHrP analogs	PTH1 receptor	Intermittent receptor activation stimulates bone formation	Peptide-based osteoanabolic scaffold	Approved	Direct anabolic activity and fracture-risk reduction	Injection route, limited treatment duration, cost	Important for severe or very-high-risk osteoporosis
Sclerostin inhibition	Wnt signaling regulation	Increases bone formation and decreases resorption	Antibody-based biologic	Approved in selected settings	Dual remodeling effect	Cardiovascular-risk evaluation required	Short-term anabolic induction in selected high-risk patients
SERMs	Estrogen receptors	Modulate estrogen-responsive skeletal and extra-skeletal pathways	Tissue-selective small-molecule ligands	Approved	Oral dosing and vertebral-fracture benefit	Tissue-specific adverse effects and limited nonvertebral efficacy	Selective use in defined patient populations
Cathepsin K inhibitors	Osteoclast protease activity	Reduces collagen matrix degradation	Small-molecule protease inhibitor scaffold	Investigational or discontinued	Mechanistically specific antiresorptive concept	Selectivity and off-target safety concerns	Illustrates translational risk in target-selective design
alphaVbeta3 integrin antagonists	Osteoclast adhesion and resorption	Interferes with osteoclast attachment to bone matrix	Small-molecule or peptidomimetic ligands	Investigational	Mechanistically rational antiresorptive approach	Modest efficacy or off-target limitations	Useful lesson for target validation and clinical endpoint selection
Natural-product-derived compounds	Multiple remodeling and inflammatory pathways	May regulate oxidative stress, inflammation, osteoblastogenesis, or osteoclastogenesis	Diverse polyphenol, flavonoid, terpenoid, alkaloid, or glycoside scaffolds	Mostly preclinical	High structural diversity and multi-target potential	Low bioavailability, batch variability, uncertain potency	Lead discovery and mechanism-probing potential
Senolytics	Senescent-cell survival pathways	Reduces senescent-cell burden or senescence-associated tissue dysfunction	Small-molecule combinations or pathway-directed agents	Early translational or preclinical in bone	Links bone aging to druggable cell states	Limited osteoporosis-specific clinical evidence	Promising but not yet established osteoporosis therapy
Bone-targeted conjugates	Bone mineral or remodeling surfaces	Increases local skeletal exposure of therapeutic cargo	Bisphosphonate, tetracycline, or oligo-aspartate targeting motifs	Preclinical to translational	May improve therapeutic index	Linker stability, release control, and biodistribution challenges	Important for potent agents with systemic toxicity risk
Nanoparticles and polymeric carriers	Bone surface, osteoclasts, osteoblasts, or marrow microenvironment	Encapsulate and release small molecules, peptides, or nucleic acids	Tunable size, surface chemistry, ligand density, and release kinetics	Preclinical	Flexible cargo and release design	Manufacturing, scale-up, immunogenicity, and reproducibility issues	Delivery platform for next-generation compounds
Engineered exosomes	Bone cells or remodeling surfaces	Delivers RNA, protein-regulating cargo, or signaling molecules	Biologically derived vesicle platform	Preclinical to early translational	Potential cell-to-cell delivery and biocompatibility	Source control, loading efficiency, purity, and potency assays	Emerging platform requiring rigorous standardization
PROTAC-related strategies	Disease-relevant proteins	Induced degradation rather than inhibition	Bifunctional degrader architecture	Emerging and mostly preclinical for bone	Expands druggable target space	Size, permeability, target selection, and safety concerns	Future discovery direction rather than current clinical therapy

## 2 Molecular mechanisms and therapeutic targets in bone remodeling

Clinically validated targets provide useful anchors for drug development, but new targets should be judged by human relevance, skeletal selectivity, druggability, safety, and evidence that modulation improves fracture-relevant outcomes rather than only biomarkers.

### 2.1 The bone remodeling cycle

Adult bone undergoes continuous remodeling by basic multicellular units (BMUs). Osteoclasts resorb bone matrix; osteoblasts subsequently deposit osteoid that mineralizes into mature bone (Frost, 1969). Under normal conditions resorption and formation are coupled in time and space. In osteoporosis the equilibrium shifts toward net resorption, producing trabecular thinning, perforation, and cortical porosity (Parfitt, 2002). Mechanistic dissection of this coupling has revealed the principal druggable nodes: the RANKL/RANK/osteoprotegerin (OPG) triad in osteoclast differentiation, the Wnt/β-catenin axis in osteoblast lineage commitment, calcium homeostasis governed by PTH and the calcium-sensing receptor (CaSR), and a set of cytokine, hormone, and matrix signals that modulate cellular activity (Baron and Kneissel, 2013; Boyle et al., 2003).

### 2.2 Osteoclastogenesis: RANKL/RANK/OPG and cathepsin K

Osteoclast precursors of monocytic lineage differentiate under the obligate influence of macrophage colony-stimulating factor (M-CSF) and RANKL. RANKL is expressed by osteoblasts, osteocytes, and stromal cells (Lacey et al., 1998). Binding of RANKL to its receptor RANK triggers TRAF6-mediated activation of NF-κB, MAPK, and NFATc1 signaling, leading to osteoclast fusion, polarization, and activation (Takayanagi, 2007). OPG, a soluble decoy receptor, binds RANKL competitively and restrains osteoclastogenesis. The mature osteoclast seals a resorption lacuna with the actin ring and secretes hydrochloric acid via the vacuolar H+-ATPase, dissolving hydroxyapatite. Cathepsin K, the dominant cysteine protease of osteoclasts, then cleaves type I collagen at the gly-pro bonds and within the triple-helix (Saftig et al., 1998). The c-Src tyrosine kinase organizes the podosome belt and the sealing zone; its pharmacological inhibition disrupts resorption while relatively sparing osteoclast survival, a feature that distinguishes Src inhibitors from bisphosphonates (Soriano et al., 1991).

### 2.3 Osteoblastogenesis: Wnt/β-catenin, BMPs, and sclerostin

Osteoblast differentiation from mesenchymal precursors is governed mainly by canonical Wnt/β-catenin signaling, with inputs from bone morphogenetic proteins (BMPs), Runx2, and Osterix (Komori, 2006). Wnt ligands bind Frizzled and low-density lipoprotein receptor-related protein 5/6 (LRP5/6), leading to stabilization and nuclear translocation of β-catenin and activation of osteogenic transcription (Krishnan et al., 2006). Two endogenous antagonists, sclerostin (encoded by SOST and produced almost exclusively by osteocytes) and Dickkopf-1 (DKK1), bind LRP5/6 competitively and inhibit Wnt signaling (Li et al., 2005). Loss-of-function mutations in SOST cause the high-bone-mass syndromes sclerosteosis and van Buchem disease. Gain-of-function LRP5 mutations cause autosomal dominant osteopetrosis (Balemans et al., 2001; Boyden et al., 2002). These human phenotypes provided the genetic rationale for sclerostin neutralization and Wnt activation as anabolic strategies (Loots et al., 2005).

### 2.4 Osteocytes as master regulators

Osteocytes are terminally differentiated osteoblasts embedded in lacuno-canalicular networks. They are the principal mechanosensors of bone, integrating mechanical strain and endocrine signals to regulate both formation and resorption (Bonewald, 2011). They produce sclerostin, RANKL, and OPG, and their viability is essential for skeletal integrity (Nakashima et al., 2011). Osteocyte apoptosis induced by estrogen deficiency or glucocorticoids contributes to disrupted remodeling (Manolagas and Parfitt, 2010). Of all the anabolic strategies attempted in the past decade, targeting osteocyte-derived sclerostin has been the most productive.

### 2.5 Hormonal regulation and emerging targets

PTH delivered intermittently is anabolic via PTH1R-mediated cAMP/PKA signaling in osteoblasts (Jilka et al., 1999). Calcitonin, estrogen, glucocorticoids, thyroid hormone, vitamin D, and IGF-1 further modulate bone turnover (Khosla et al., 2012). Recent attention has focused on activin and myostatin signaling. These TGF-β superfamily ligands engage type II activin receptors (ActRIIA/B) and negatively regulate bone and muscle mass. Soluble decoy receptors and neutralizing antibodies have produced skeletal anabolic effects (Pearsall et al., 2008). The CaSR, a class C G-protein-coupled receptor, regulates endogenous PTH secretion. Antagonists known as calcilytics induce a transient PTH pulse intended to mimic the pharmacokinetics of teriparatide (Nemeth et al., 2004). Other targets of current interest include EphrinB2/EphB4, Semaphorin 3A, Schnurri-3, RANKL reverse signaling, and the bone marrow adipocyte secretome (Hayashi et al., 2012; Zhao et al., 2006).

## 3 Established pharmacotherapies and limitations

The greatest value of novel chemical entities lies in addressing well-defined unmet needs that current approved therapies fail to satisfy—specifically, achieving skeletal selectivity, enabling oral anabolic activity, ensuring improved long-term safety, enhancing patient access, or facilitating compatibility with sequential treatment regimens.

### 3.1 Bisphosphonates

Bisphosphonates are stable P–C–P pyrophosphate analogues. They bind hydroxyapatite with high affinity and are internalized by osteoclasts at sites of active resorption (Russell, 2011). Nitrogen-containing bisphosphonates inhibit farnesyl pyrophosphate synthase (FPPS) in the mevalonate pathway. This blocks prenylation of the small GTPases Rho, Rac, and Rab that are essential for osteoclast cytoskeletal organization, and triggers osteoclast apoptosis (van Beek et al., 1999). Pivotal trials, including FIT for alendronate, VERT for risedronate, and HORIZON for zoledronic acid, demonstrated 40%–70% reductions in vertebral fracture risk and 20%–40% reductions in non-vertebral and hip fractures (Black et al., 1996; Black et al., 2007). Gastrointestinal intolerance limits the use of oral agents. Intravenous zoledronate produces an acute-phase reaction. Prolonged exposure has been linked to atypical femoral fractures and osteonecrosis of the jaw (Whitaker et al., 2012). The half-life of the drug in bone is measured in years, which complicates decisions about drug holidays.

### 3.2 Denosumab

Denosumab is a fully human IgG2 monoclonal antibody against RANKL. It mimics endogenous OPG and dose-dependently suppresses osteoclastogenesis (Kostenuik et al., 2009). In the pivotal FREEDOM trial, denosumab 60 mg subcutaneously every 6 months reduced vertebral fractures by 68%, hip fractures by 40%, and non-vertebral fractures by 20% over 3 years (Cummings et al., 2009). Ten-year extension data confirmed sustained efficacy with progressive BMD gains (Bone et al., 2017). However, denosumab discontinuation produces a rapid rebound in bone turnover, with rapid BMD loss and a documented risk of multiple vertebral fractures within 12 months. Sequential therapy with a bisphosphonate is therefore required (Cummings et al., 2018).

### 3.3 SERMs and hormone therapy

Raloxifene and bazedoxifene act as estrogen receptor agonists in bone and antagonists in breast tissue. They reduce vertebral fracture risk by approximately 30%–40% without endometrial proliferation (Cummings et al., 2010). They do not reduce non-vertebral fractures meaningfully and carry venous thromboembolism risk. The combination of conjugated estrogen and bazedoxifene, known as the tissue-selective estrogen complex, provides menopausal symptom relief with bone protection (Pinkerton et al., 2014).

### 3.4 PTH and PTHrP analogues

Teriparatide (PTH 1–34) and abaloparatide, a PTHrP analogue, are given once daily by subcutaneous injection. Both produce a transient PTH1R-mediated anabolic response with significant increases in spine BMD and reductions in vertebral and non-vertebral fractures (Black et al., 2003; Leder et al., 2015). The ACTIVE trial reported that abaloparatide reduced vertebral fractures by 86% and non-vertebral fractures by 43% versus placebo over 18 months (Miller et al., 2016). Use is limited to 24 months of cumulative treatment because of an early rodent osteosarcoma signal. The signal has subsequently been considered species-specific and a human risk has not been confirmed (Vahle et al., 2002). Sequential antiresorptive therapy is required to consolidate the gains.

### 3.5 Romosozumab

Romosozumab is a humanized anti-sclerostin IgG2 antibody. It stimulates bone formation and suppresses resorption at the same time. This uncoupled pharmacology produces the largest 1-year BMD gains of any current therapy (Padhi et al., 2011). The FRAME trial reported a 73% reduction in new vertebral fractures at 12 months versus placebo (Cosman et al., 2016). The ARCH trial demonstrated superiority over alendronate in postmenopausal women with prior fracture (Saag et al., 2017). A cardiovascular imbalance observed in ARCH, with more myocardial infarctions and strokes in the romosozumab arm, led to regulatory cautions and a contraindication in patients with recent cardiovascular events (Bovijn et al., 2020).

### 3.6 Persistent gaps

Several gaps remain. No oral osteoanabolic agent has been approved. The long-term safety of multi-decade antiresorptive exposure is not fully characterized. Rebound phenomena complicate sequencing. Non-vertebral fracture reductions plateau at around 20%–40% across most agents. The patient experience, with injections, gastrointestinal restrictions, and monitoring, contributes to poor adherence (Black and Rosen, 2016). These limitations frame the rationale for the compounds discussed in the rest of this review.

## 4 Chemistry-driven discovery of novel osteoanabolic compounds

Osteoanabolic drug discovery must evolve beyond mere cataloguing of bioactivity. Future efforts should prioritize elucidating exposure–response relationships, refining target selectivity, and validating durable skeletal benefits—particularly the clinical feasibility of initiating therapy with a potent anabolic induction phase followed by a cost-effective maintenance regimen.

### 4.1 Sclerostin pathway: from biologics to small molecules

Sclerostin biology has been one of the most productive translational stories in skeletal disease over the past 2 decades. The clinical molecule, romosozumab, is nevertheless an injectable antibody. Medicinal chemistry has therefore pursued small-molecule and peptide mimetics of antibody activity. The principal binding site is the sclerostin loop2 region responsible for LRP5/6 engagement (Glorieux et al., 2017). Cyclic peptides identified by phage display have shown nanomolar affinity for sclerostin and have recapitulated Wnt pathway activation *in vitro*. Optimization for proteolytic stability and bioavailability is ongoing. Blosozumab and setrusumab are additional anti-sclerostin antibodies; setrusumab is currently in trials for osteogenesis imperfecta (Recker et al., 2015). Beyond antibodies and peptidomimetics, anti-sclerostin aptamers and small-molecule Wnt agonists targeting GSK-3β, axin, or disruption of the β-catenin–Tcf4 interaction have been described. The principal chemistry challenge is selectivity over off-target tumorigenic Wnt activation (Bourhis et al., 2010; Krishnamurthy and Kurzrock, 2018).

### 4.2 DKK1 inhibitors

DKK1 is the second principal endogenous LRP5/6 antagonist. It is upregulated in multiple myeloma–associated bone disease, in glucocorticoid-induced osteoporosis, and in rheumatoid arthritis (Tian et al., 2003). The neutralizing antibody BHQ880 has shown anabolic activity in myeloma bone disease in early-phase trials (Iyer et al., 2014). Bispecific antibodies and engineered scaffolds targeting sclerostin and DKK1 together have shown superior anabolic efficacy in preclinical models. Dual targeting offers a rational way around the compensatory upregulation seen with single-target inhibition (Florio et al., 2016).

### 4.3 PTH/PTHrP analogues and orally bioavailable PTH1R agonists

Beyond teriparatide and abaloparatide, the long-acting PTH/PTHrP hybrid PL-3994 and PTH1R-biased agonists that selectively engage Gαs over β-arrestin pathways have been studied for their effect on the anabolic-to-resorptive ratio (Hattersley et al., 2016). Oral small-molecule PTH1R agonists are of particular interest. PCO371, a non-peptide PTH1R-selective molecule, achieved oral bioavailability and bone anabolic effects in preclinical species. It is in early clinical development for hypoparathyroidism (Vilardaga and Heredero-Jimenez, 2026). PCO371 challenges the long-held view that class B GPCRs cannot be addressed by small molecules. Structure-based design that exploits cryo-EM structures of activated receptor complexes was central to its discovery (Zhao et al., 2019).

### 4.4 Calcium-sensing receptor antagonists (calcilytics)

Calcilytics are small-molecule allosteric antagonists of CaSR. They induce a rapid, transient endogenous PTH pulse intended to mimic the pharmacokinetic profile of injected teriparatide (Nemeth and Goodman, 2016). Compounds including ronacaleret, MK-5442, and JTT-305/MK-5442 reached phase II–III trials. None matched alendronate in head-to-head comparisons, which underscores the difficulty of reproducing peptide hormone pharmacology with orally absorbed lipophilic compounds (Halse et al., 2014). The chemotype illustrates that allosteric modulation of class C GPCRs is feasible. Re-engineered calcilytics remain of interest for hypocalcemia in autosomal dominant hypocalcemia type 1 (Hannan et al., 2018).

### 4.5 Activin/myostatin pathway modulators

Activin A and myostatin (GDF-8) are members of the TGF-β superfamily. They bind ActRIIA/B receptors and inhibit muscle and bone mass (Lee and McPherron, 2001). Sotatercept (ActRIIA-Fc) and bimagrumab (anti-ActRII antibody) have been tested in muscle wasting and bone disease. Sotatercept produced increases in lumbar BMD in patients with end-stage renal disease and chronic anemia (Sherman et al., 2013). Luspatercept, a related ligand trap approved for transfusion-dependent anemias in β-thalassemia and myelodysplastic syndromes, has secondary effects on bone metabolism that have not been systematically characterized (Cappellini et al., 2020). Small-molecule ALK4/5/7 kinase inhibitors face selectivity challenges against the broader TGF-β receptor family.

### 4.6 Anti-schnurri-3 and miscellaneous anabolic targets

Schnurri-3 (SHN3) is an E3 ubiquitin ligase that promotes Runx2 degradation. SHN3 deletion in mice yields a striking high-bone-mass phenotype. Anti-SHN3 antibodies engineered for selective osteoblast delivery via Asp8-tagged carriers have been described (Yang et al., 2015). Loss-of-function studies of LGR4, a Wnt amplifier, of SFRP1, and miR-214 antagomirs likewise point to anabolic chemistry opportunities (Luo et al., 2016; Wang et al., 2013).

## 5 Novel antiresorptive small molecules

Antiresorptive research benefits from a solid foundation of validated biological pathways; however, the field is saturated with candidates that offer little beyond a novel target or enhanced *in vitro* activity. True progress demands a demonstrable improvement in the therapeutic index to justify their clinical development. For drug development, the most viable path for novel antiresorptives lies in improving the therapeutic index of existing mechanisms and in extending disease-modifying benefits beyond classical osteoporosis, while guarding against the off-target liabilities that curtailed prior programmes.

### 5.1 Cathepsin K inhibitors

Cathepsin K (CTSK) is the dominant osteoclastic cysteine protease. It cleaves type I collagen within the triple-helical region. Genetic deficiency causes pycnodysostosis, characterized by dense but brittle bones, a phenotypic warning to chemists (Gelb et al., 1996). Several chemotypes have been developed. These include nitrile-based reversible covalent inhibitors such as odanacatib, aldehyde- and ketone-based reversible inhibitors, and macrocyclic peptidomimetics (Bromme and Lecaille, 2009). Odanacatib (MK-0822) was a once-weekly oral dose. It produced robust BMD gains and a 54% reduction in hip fractures in the LOFT trial (McClung et al., 2019). Development was halted because of a numerically increased risk of stroke in the active arm (Mullard, 2016). The cardiovascular signal has been attributed to off-target effects of accumulating cathepsin K outside bone, or to off-target inhibition of cathepsins B, L, or S. Subsequent compounds (ONO-5334, MIV-711, ODN-related analogues) have been re-engineered to address selectivity, tissue distribution, and reversibility (Nagase et al., 2012). MIV-711 has shown disease-modifying effects in osteoarthritis trials, suggesting a role for cathepsin K inhibitors beyond classical osteoporosis (Conaghan et al., 2020). The lessons from this chemotype are instructive. Selectivity over cathepsins B/L/S is essential. Reversible inhibition with a fast off-rate minimizes off-skeletal effects (Lindstrom et al., 2018).

### 5.2 Selective androgen receptor modulators (SARMs)

SARMs are non-steroidal small molecules with tissue-selective androgen receptor agonism. They were originally developed for sarcopenia and cachexia. Enobosarm (GTx-024, ostarine) and LGD-4033 increase lean mass and have produced modest BMD increases in animal models and exploratory trials (Dalton et al., 2011). The chemistry challenge is achieving anabolic selectivity in muscle and bone without effects on the prostate or liver. Long-term oncologic risk remains under scrutiny (Solomon et al., 2019).

### 5.3 SERMs of the next-generation

Lasofoxifene and ospemifene extend the SERM franchise. Lasofoxifene reduced vertebral and non-vertebral fractures with improved venous safety in postmenopausal cohorts. Subsequent regulatory paths and oncology applications have driven its development (Goldstein et al., 2014). The medicinal chemistry of SERMs continues to refine the balance between tissue-specific ER agonism and antagonism via subtle modifications of the triarylethylene core (Komm and Mirkin, 2014).

### 5.4 Src and c-Fms inhibitors

Saracatinib (AZD0530) is a dual Src/Abl inhibitor. It suppressed bone resorption in clinical pharmacology studies, but development for osteoporosis was discontinued in favor of oncology applications (Hannon et al., 2010). The colony-stimulating factor 1 receptor (CSF1R, c-Fms) inhibitor PLX3397 (pexidartinib) blocks osteoclast precursor recruitment. It is approved for tenosynovial giant cell tumor, although hepatotoxicity has limited broader bone applications (Tap et al., 2015).

### 5.5 Integrin αvβ3 antagonists and chloride channel modulators

The αvβ3 integrin is the dominant adhesion molecule mediating osteoclast attachment to bone matrix. Cyclic RGD peptidomimetics and non-peptide small molecules showed transient antiresorptive activity in early bone studies, but proof of fracture reduction has not been delivered (Engleman et al., 1997). ClC-7 chloride channel modulators, which intersect with osteopetrosis biology, represent a more speculative chemistry frontier (Kornak et al., 2001).

## 6 Natural products and their chemically optimized derivatives

Natural products have provided a continuing source of bone-active chemotypes. Many reflect centuries of empirical use in traditional medical systems. Contemporary medicinal chemistry has progressed from crude extracts to isolated pharmacophores, semi-synthetic derivatives, and rationally optimized analogues with improved potency, selectivity, and bioavailability.

Collectively, natural-product-derived chemotypes remain most valuable as lead-discovery platforms, with clinical translation dependent on successful delivery optimization to overcome persistent bioavailability and standardization barriers.

### 6.1 Flavonoids

#### 6.1.1 Icariin and icaritin

Icariin is a prenylated flavonoid glycoside from *Epimedium* species and has been used in Chinese herbal therapy for skeletal complaints for centuries. It enhances osteoblastic differentiation and inhibits osteoclastogenesis through activation of BMP-2/Smad and Wnt signaling and through suppression of NF-κB (Zhang et al., 2010). Icaritin, the deglycosylated aglycone, has better membrane permeability and oral bioavailability. Semi-synthetic prenyl analogues have advanced into clinical trials, including for hepatocellular carcinoma and bone metabolic disorders (Wu et al., 2017). SAR studies indicate that the 8-prenyl group and the 4′-methoxy substitution are critical to osteogenic activity. The C3 sugar diminishes potency (Wei et al., 2016).

#### 6.1.2 Genistein and isoflavones

Soy-derived genistein is a phytoestrogen acting principally via ERβ. Meta-analyses of clinical trials support modest BMD preservation in postmenopausal women, although fracture data are absent (Marini et al., 2007). The 4′,5,7-trihydroxyisoflavone scaffold supports extensive derivatization. Methylated, glycosylated, and prenylated analogues, including 8-prenyl genistein and formononetin, have shown improved osteogenic effects in preclinical models (Pan et al., 2005).

#### 6.1.3 Naringin, naringenin, kaempferol, quercetin

Naringin, the principal flavanone glycoside of *Citrus* and *Drynaria*, promotes osteoblast differentiation and inhibits osteoclastogenesis at micromolar concentrations (Wong and Rabie, 2006). Its aglycone naringenin is more permeable but more rapidly conjugated. Kaempferol and quercetin, both ubiquitous flavonols, modulate Wnt and AMPK signaling. Quercetin also features prominently in senolytic combinations (Section 7; Wong et al., 2020). B-ring hydroxylation patterns and prenylation profoundly affect bone activity and provide a rich landscape for SAR exploration.

### 6.2 Stilbenes and polyphenols

#### 6.2.1 Resveratrol

trans-3,5,4′-Trihydroxystilbene, classically associated with red wine, activates SIRT1, AMPK, and ER signaling and modulates osteoblast and osteoclast function (Mobasheri and Shakibaei, 2013). Encouraging preclinical data have not translated cleanly into postmenopausal clinical trials, where BMD effects have been inconsistent. The principal reason is poor oral bioavailability of less than 1% (Ornstrup et al., 2014). Semi-synthetic derivatives, including pterostilbene (the dimethyl ether) and methylated analogues, improve absorption and have shown stronger osteoblast-promoting effects *in vitro* (Riche et al., 2014).

#### 6.2.2 Curcumin and analogues

Curcumin is the principal diferuloylmethane of *Curcuma longa*. It exerts anti-inflammatory and osteoprotective effects via NF-κB inhibition and Nrf2 activation (Aggarwal and Harikumar, 2009). Its near-zero oral bioavailability has driven an extensive medicinal chemistry effort. The output includes curcumin-phospholipid complexes, nanoformulations, prodrugs, and synthetic analogues such as EF-24, GO-Y030, and CDF (difluorinated curcumin) with much improved pharmacokinetics (Anand et al., 2007).

#### 6.2.3 Epigallocatechin gallate (EGCG)

EGCG is the principal catechin of green tea. It inhibits RANKL-induced osteoclastogenesis through suppression of NF-κB and JNK and promotes osteoblastic differentiation (Lin et al., 2018). Rapid metabolism and methylation limit clinical translation. Combinations with proline-rich peptides and encapsulation strategies have been explored (Shen et al., 2009).

### 6.3 Triterpenoids and saponins

#### 6.3.1 Oleanolic and ursolic acids

These pentacyclic triterpenoids show dual anabolic and antiresorptive effects, partly via Wnt activation and partly via RANKL suppression (Nogueira-Júnior et al., 2024). Synthetic derivatives including 2-cyano-3,12-dioxooleana-1,9(11)-dien-28-oic acid (CDDO) and bardoxolone-class Nrf2 activators have potent anti-inflammatory effects relevant to bone preservation in chronic inflammatory states (Liby and Sporn, 2012).

#### 6.3.2 Ginsenosides and astragalosides

Triterpene saponins from *Panax ginseng* and *Astragalus membranaceus* support osteoblastic differentiation *in vitro* and bone preservation in ovariectomized rats. Ginsenosides Rg1, Rb1, Rh2, and CK have been studied as scaffolds for further optimization (Siddiqi et al., 2013). Astragaloside IV has shown anti-osteoclastogenic activity via modulation of the RANKL/OPG ratio (Yang et al., 2014).

### 6.4 Coumarins and other natural scaffolds

#### 6.4.1 Psoralen and isopsoralen

These furocoumarins from *Psoralea corylifolia* have long been used in traditional Chinese medicine. They activate ERα and ERβ and stimulate osteoblastic differentiation through BMP/Smad signaling (Wang et al., 2011). Hepatotoxicity has been a recurring concern, and chemistry effort has focused on detoxification of the furocoumarin core (Tang et al., 2010).

#### 6.4.2 Berberine

This isoquinoline alkaloid from *Coptis chinensis* inhibits osteoclastogenesis and promotes osteoblastic differentiation. Oral bioavailability is poor but is improved by nanoformulation (Vilardaga and Heredero-Jimenez, 2026).

#### 6.4.3 Salvianolic acid B

The principal water-soluble bioactive of *Salvia miltiorrhiza*, salvianolic acid B activates BMP-2 expression and AMPK signaling and has produced bone-protective effects in ovariectomized and glucocorticoid-induced osteoporosis models (Cui et al., 2009).

Cardamonin, hesperidin, formononetin, calycosin, baicalein, wogonin, and many other natural flavonoids and chalcones are at various preclinical stages, often as adjuncts in poly-herbal formulations such as Er-Xian decoction and the Bu-Shen-Huo-Xue formulas, whose bioactive components are increasingly characterized at the molecular level (Wang et al., 2025).

Several chemistry challenges recur across natural-product programs. These include poor aqueous solubility, rapid metabolism, low oral bioavailability, and structural complexity that complicates scale-up. They drive ongoing innovation in semi-synthesis, prodrug design, and formulation. Regulatory pathways for botanical drugs are evolving, and natural-product chemistry is increasingly recognized as a legitimate route to fully characterized therapeutic agents rather than a relic of pre-rational pharmacology (Xu et al., 2019).

## 7 Senolytics and bone aging

Cellular senescence is a state of stable cell-cycle arrest accompanied by a pro-inflammatory secretome known as the senescence-associated secretory phenotype (SASP). Senescent osteocytes, osteoblasts, and bone-marrow stromal cells accumulate with age and after irradiation or chemotherapy. They contribute to bone loss (Farr et al., 2017). Senolytics are small molecules that selectively kill senescent cells by disrupting their anti-apoptotic SCAP pathways. They have become a chemistry-driven anti-aging strategy applicable to skeletal disease (Kirkland and Tchkonia, 2020).

The prototypical combination of the tyrosine kinase inhibitor dasatinib (D) with the flavonoid quercetin (Q) targets distinct senescent-cell anti-apoptotic networks. The pair reduced senescent cell burden in aged mice with parallel improvements in trabecular bone microarchitecture (Zhu et al., 2015). Fisetin, a structurally related flavonol with single-agent senolytic activity, has progressed into multiple human trials (Yousefzadeh et al., 2018). Navitoclax (ABT-263) is a Bcl-2/Bcl-xL/Bcl-w inhibitor with potent senolytic activity but dose-limiting thrombocytopenia. Structural derivatives such as ABT-737 and Bcl-xL-selective PROTAC degraders (for example, DT2216) reduce platelet toxicity and represent an emerging chemistry direction (Khan et al., 2019). Other senolytic chemotypes include HSP90 inhibitors, FOXO4-DRI peptides, and the natural products piperlongumine and procyanidin C1 (Fuhrmann-Stroissnigg et al., 2017).

Translational data in human osteoporosis are preliminary. Early clinical pharmacology trials in older adults are documenting feasibility and target engagement. Well-designed fracture-endpoint trials remain a future imperative (Hickson et al., 2019). While mechanistically compelling in the context of bone aging, senolytic strategies should be framed as emerging therapeutics. Their clinical translation remains contingent upon rigorous osteoporosis-specific evaluations of safety, dose optimization, target engagement, and definitive efficacy.

For clinical development, senolytic strategies are presently best suited to hypothesis-generating lead discovery and biomarker-driven proof-of-concept studies rather than immediate maintenance therapy, pending osteoporosis-specific fracture-endpoint evidence.

## 8 Bone-targeted drug delivery

Directing potent compounds preferentially to bone has been a persistent challenge across osteoporosis pharmacology. The goal is to deliver pharmacology where it is needed while minimizing systemic exposure. Bisphosphonate-drug conjugates exploit the high affinity of P–C–P pyrophosphate scaffolds for hydroxyapatite (Hirabayashi and Fujisaki, 2003). The estrogen-bisphosphonate conjugate ER-NS illustrates the principle, achieving selective bone-targeted estrogen action with minimized extra-skeletal exposure (Houghton et al., 2008). Tetracycline and oligo-aspartate moieties offer alternative bone-recognition handles (Zhang et al., 2012).

Nanoparticle delivery, including liposomes, polymeric nanoparticles, exosomes, and lipid nanoparticles, has been engineered with bone-homing peptides such as (DSS)6, (AspSerSer)6, and Asp8 (Wang et al., 2018). siRNA delivery to osteoclasts (for example, targeting cathepsin K or NFATc1) and to osteoblasts (for example, delivering miRNA antagomirs against miR-214) has been demonstrated in rodent osteoporosis models (Liu et al., 2015). Bone-targeted carrier chemistry, from biodegradable polymer backbones to surface-functionalized exosomes, represents an active area at the interface of materials science and medicinal chemistry (Cui et al., 2022).

Beyond pharmacokinetic targeting, bone-targeted PROTACs and antibody-drug conjugates with bisphosphonate or tetracycline linkers offer the possibility of locally restricted protein degradation. This is an emerging modality with theoretically minimized systemic risk (Wells and Kumru, 2024).

## 9 From preclinical validation to clinical translation: challenges and strategies

Clinical attrition of bone-active compounds is striking. Of the candidates entering phase I development for osteoporosis between 1990 and 2020, fewer than 15% reached regulatory approval. Several promising classes (cathepsin K inhibitors, calcilytics, integrin antagonists, Src inhibitors) failed for reasons that ranged from off-target safety to insufficient efficacy in human bone physiology (Hopkins, 2008). Robust translational success hinges on the congruence of mechanistic rationale, chemical design, systemic exposure, safety margins, preservation of bone quality, and clinically meaningful endpoints.

### 9.1 Pharmacokinetic optimization

Bone is a poorly perfused mineralized tissue. Many lipophilic small molecules concentrate poorly there. Strategies to address this include bisphosphonate prodrug conjugation, nanoparticle delivery, and physicochemical optimization to favor mineral binding (Stresing et al., 2007). Oral bioavailability is a recurring weak point for natural products and peptidomimetics. Macrocyclization, N-methylation, and lipid-based formulations have all been deployed (Kratz et al., 2008).

### 9.2 Species differences in bone biology

Ovariectomized rats and mice remain the workhorses of preclinical osteoporosis pharmacology. Rodent bone, however, differs from human bone in remodeling rates, cortical microstructure, and Haversian remodeling. Larger species (rabbit, dog, non-human primate) better recapitulate human cortical biology but introduce ethical and logistic constraints (Reinwald and Burr, 2008). The teriparatide rodent osteosarcoma signal, which proved species-specific, illustrates the bidirectional challenges of cross-species translation (Andrews et al., 2012).

### 9.3 Large samples and long durations

Osteoporosis trials are demanding. Annual fracture incidence is low, so large samples and long durations are required. Surrogate endpoints such as BMD and bone turnover markers are well-validated but imperfect (Bouxsein et al., 2019). Adaptive designs, fracture-risk-enriched cohorts (for example, recent prior fracture), and active-comparator designs rather than placebo increasingly characterize modern trials (Eastell and Szulc, 2017). The ARCH trial of romosozumab versus alendronate is a landmark example (Langdahl et al., 2017).

### 9.4 Safety pharmacology and biomarkers

Retrospective recognition of cardiovascular signals with romosozumab and odanacatib has emphasized the need for early and systematic cardiovascular safety assessment in bone-active compounds (Cummings and McCulloch, 2020). Bone turnover markers including CTX, P1NP, and osteocalcin are valuable pharmacodynamic biomarkers but do not capture site-specific or qualitative effects on bone material properties (Vasikaran et al., 2011). Advanced imaging such as high-resolution peripheral quantitative computed tomography (HR-pQCT), micro-MRI, and finite-element analysis increasingly complements DXA in clinical pharmacology (Boutroy et al., 2005).

### 9.5 Long-term outcomes and sequencing

Most osteoporosis pharmacotherapy is a multi-decade endeavor. Questions of optimal sequencing, including anabolic-first versus antiresorptive-first regimens, switching strategies, and drug holidays, are increasingly central (Tsai et al., 2013). Anabolic-first regimens, in which teriparatide or romosozumab is followed by an antiresorptive, produce greater BMD gains than the reverse sequence. This finding has reshaped clinical practice and informed regulatory labels (Cosman et al., 2017).

## 10 Future perspectives

The next-generation of bone therapeutics demands a convergent paradigm—one that synergizes advanced computation with hypothesis-driven medicinal chemistry, precision bone-targeted delivery, and validation pipelines anchored in human biology and clinically relevant outcomes.

### 10.1 Artificial intelligence-guided drug discovery

Machine learning and deep generative models are accelerating bone-active compound discovery. Applications range from ligand-based virtual screening of natural-product libraries to structure-based optimization using cryo-EM structures of activated PTH1R, CaSR, and Frizzled complexes (Jumper et al., 2021). Bone-targeted PROTACs designed *in silico* are beginning to populate the literature, drawing on AlphaFold-derived ternary complex models.

### 10.2 PROTACs and molecular glues

Targeted protein degradation by PROTACs and molecular glues offers a different mode of action: catalytic, event-driven degradation rather than stoichiometric inhibition (Bekes et al., 2022). Bone-relevant targets including sclerostin, DKK1, cathepsin K, ER, and RANK have all been proposed for degrader development. The principal chemistry challenges are E3 ligase selection (cereblon, VHL, IAP), linker design, and tissue distribution.

### 10.3 Combination and personalized regimens

Genetic risk stratification using polygenic risk scores for BMD and fracture, together with pharmacogenomic data, is beginning to inform individual treatment selection (Estrada et al., 2012). Combination regimens, including denosumab plus teriparatide and the sequential romosozumab-to-denosumab approach, yield BMD gains superior to monotherapy. The chemistry of co-formulation and of dual-mechanism single molecules remains an open frontier (Leder et al., 2014).

### 10.4 Special populations

Pediatric osteoporosis, osteogenesis imperfecta, glucocorticoid-induced osteoporosis, transplant-related bone disease, and male osteoporosis represent populations with distinct biology and chemistry needs (Bishop et al., 2014). Setrusumab in osteogenesis imperfecta and burosumab in X-linked hypophosphatemia illustrate the increasingly individualized deployment of bone-active biologics (Carpenter et al., 2018).

## 11 Limitations

This review is broad and chemistry-oriented. As a result, it cannot discuss every clinical guideline, epidemiological factor, animal model, biomarker, or mechanistic pathway in full depth. The review also includes therapeutic areas at different stages of maturity, from approved drugs to early preclinical concepts. To avoid overinterpretation, investigational areas such as senolytics, PROTAC-related strategies, AI-guided discovery, and personalized regimens are presented as emerging directions unless osteoporosis-specific clinical evidence is available. The objective is to provide a chemistry-driven translational framework rather than a complete clinical practice guideline.

## 12 Conclusion

The chemistry-driven discovery pipeline for osteoporosis has changed substantially over the past 2 decades. The field has moved from the canonical bisphosphonate scaffold to monoclonal antibodies against RANKL and sclerostin, and is now exploring oral small-molecule PTH1R agonists, senolytic combinations, and bone-targeted PROTAC degraders. Several notable failures have been instructive. Odanacatib, the calcilytics, and the integrin antagonists all illustrate how off-target activity and species-specific pharmacology can derail otherwise convincing programs. The successes are equally instructive. Romosozumab in particular illustrates how human genetic evidence, careful structural biology, and adequately powered clinical trials can translate a single-gene discovery into a registered therapeutic.

Important questions remain open. No oral osteoanabolic agent has yet reached approval, the long-term safety of multi-decade osteoporosis therapy remains incompletely characterized, and many natural-product chemotypes with interesting biology continue to stall because of bioavailability, standardization, or pharmacokinetic limitations. Global access to established therapies also remains uneven. Addressing these gaps will require sustained investment in human target validation, SAR-driven medicinal chemistry, skeletal selectivity, bone-targeted delivery platforms, and adequately powered clinical trials with fracture-relevant endpoints. By linking synthesis, mechanism, drug-like properties, delivery strategy, and translational evaluation, future research can more efficiently move promising bioactive compounds from preclinical validation toward clinically useful osteoporosis therapy.
